# Laboratory breeding of two *Phortica* species (Diptera: Drosophilidae), vectors of the zoonotic eyeworm *Thelazia callipaeda*

**DOI:** 10.1186/s13071-022-05331-6

**Published:** 2022-06-13

**Authors:** Ilaria Bernardini, Cristiana Poggi, Sara Manzi, Marcos Antonio Bezerra-Santos, Frédéric Beugnet, Josephus Fourie, Domenico Otranto, Marco Pombi

**Affiliations:** 1 Dipartimento Di Malattie Infettive, Istituto Superiore Di Sanità, Rome, Italy; 2 Dipartimento Di Sanità Pubblica E Malattie Infettive, Sapienza Università Di Roma, Rome, Italy; 3 Dipartimento Di Medicina Veterinaria, Università Degli Studi Di Bari, Valenzano, Italy; 4 Boehringer-Ingelheim Animal Health, Lyon, France; 5 ClinVet International (Pty) Ltd, Bloemfontein, South Africa

**Keywords:** *Phortica oldenbergi*, *Phortica variegata*, *Phortica semivirgo*, *Thelazia callipaeda*, Eyeworm, Laboratory rearing, Vector-borne disease, Zoonosis

## Abstract

**Background:**

Some species of drosophilid flies belonging to the genus *Phortica* feed on ocular secretions of mammals, acting as biological vectors of the zoonotic eyeworm *Thelazia callipaeda*. This study describes an effective breeding protocol of *Phortica variegata* and *Phortica oldenbergi* in insectary conditions.

**Methods:**

Alive gravid flies of *P. oldenbergi*, *P. variegata* and *Phortica semivirgo* were field collected in wooded areas of Lazio region (Italy) and allowed to oviposit singularly to obtain isofamilies. Flies were maintained in ovipots (200 ml) with a plaster-covered bottom to maintain high humidity level inside. Adult feeding was guaranteed by fresh apples and a liquid dietary supplement containing sodium chloride and mucin proteins, while larval development was obtained by *Drosophila*-like agar feeding medium. The breeding performances of two media were compared: a standard one based on cornmeal flour and an enriched medium based on chestnut flour. All conditions were kept in a climatic chamber with a photoperiod of 14:10 h light:dark, 26 ± 2 °C and 80 ± 10% RH.

**Results:**

From a total of 130 field-collected *Phortica* spp., three generations (i.e. F1 = 783, F2 = 109, F3 = 6) were obtained. *Phortica oldenbergi* was the species with highest breeding performance, being the only species reaching F3. Chestnut-based feeding medium allowed higher adult production and survival probability in both *P. oldenbergi* and *P. variegata*. Adult production/female was promising in both species (*P. oldenbergi*: 13.5 F1/f; *P. variegata*: 4.5 F1/f).

**Conclusions:**

This standardized breeding protocol, based on controlled climatic parameters and fly densities, together with the introduction of an enriched chestnut-based feeding medium, allowed to investigate aspects of life history traits of *Phortica* spp. involved in the transmission of *T. callipaeda*. Obtaining F3 generation of these species for the first time paved the road for the establishment of stable colonies, an essential requirement for future studies on these vectors in controlled conditions.

**Graphical abstract:**

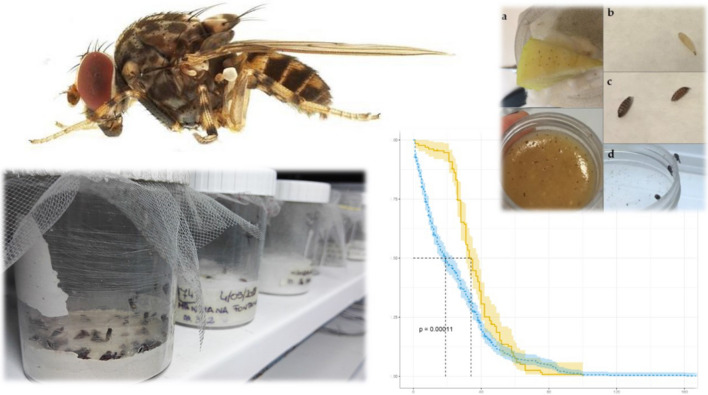

## Background

The *Phortica* genus (Diptera: Drosophilidae) includes around 130 species distributed worldwide, especially in mountain areas of the Oriental Region, where many species occur [1]. Most of the species of the subgenus *Phortica* (which includes *P. variegata*, *P. foliiseta P. magna* and *P. omega* complexes, and *P. varipes* group) have adapted to feeding on ocular secretions of mammals [2–5]. In particular, four lachryphagous species, *Phortica variegata, Phortica okadai*, *Phortica magna* and *Phortica kappa*, have been identified as intermediate hosts and vectors of the zoonotic eyeworm *Thelazia callipaeda* (Railliet & Henry; Spirurida: Thelaziidae) [3, 6, 7]. This eyeworm infests the eye of dogs, cats, wild carnivores (e.g. foxes, wolves, bears), lagomorphs, and humans in Europe and Asia [8, 9]. Currently, several cases of human thelaziosis by *T. callipaeda* have been reported in Asia (i.e. China, Korea, India, Thailand and Japan) and Europe, with an increasing trend in recent decades [8, 10, 11]. Recently, an additional species, *Phortica oldenbergi*, has been experimentally demonstrated as intermediate host of *T. callipaeda* (Bezerra Santos et al., submitted). To date, four *Phortica* spp. (i.e. *Phortica erinacea*, *P. oldenbergi*, *Phortica semivirgo*, and *P. variegata*) have been identified in Europe [12], with *P. variegata* being the most prevalent species in many regions [13]. Conversely, for the other species no information is available about their natural life history, pre- and post-mating behaviour and ecology [1].

It is of paramount importance to breed colonies of arthropods in controlled conditions. From the early 1900s to now, breeding of several invertebrate taxa (e.g. moths, mosquitoes, beetles, marine copepods and fruit flies) has been pivotal to study subjects in many fields of science, including evolution, ecology and physiology [14]. In medical and veterinary contexts, the establishment of arthropod colonies is pivotal to investigating their life cycles, genetics, behaviour, interaction with vector-borne parasites and vector competence or susceptibility to insecticides [15]. Several rearing protocols have been developed for studying vectors of medical and veterinary concern such as mosquitoes, sand flies, tsetse flies [16–18] and *P. variegata*, for which a single protocol has been described in literature [19].

This study aims to describe a novel, standardized rearing method for *P. variegata* and *P. oldenbergi* flies, based on an artificial diet and characterized parameters of density and climatic conditions suitable to create a potentially stable colony. Results will represent an important starting point for controlled studies on *Phortica* spp. life cycle and vector role, toward in vitro testing of new insecticidal drugs and reducing the vector capacity of these drosophilids.

## Methods

### Sample collection

*Phortica* spp. gravid females were collected in Manziana (Lazio region, Italy, 42°07′09″N, 12°06′58″E; altitude 378 m a.s.l.) from May to September 2020. Wild females were collected with an entomological net around a bait of decaying fruits (i.e. apples, bananas, peaches, pears), which was placed into a cloth tied with a string around the bark of Turkey oak trees (*Quercus cerris*). Specimens were subsequently identified as *P. oldenbergi, P. semivirgo* and *P. variegata* using identification keys [18]. Gravid females were identified based on the enlarged abdomen and a yellowish colour on the abdominal sternites. Later on, the flies were individually transferred into plastic pots and immediately transported alive to the laboratory at the Department of Public Health and Infectious Diseases, Sapienza University of Rome (Italy). During the travel, pieces of fresh apple were added to the pots as food source and the containers were placed in a plastic box with a moist cloth pad to maintain high humidity and to protect flies from sunlight and excessive heat.

### Rearing conditions

A maximum of ten female specimens was reared in 200-ml (8 cm height, 5.5 cm diameter) cylindric transparent plastic containers (oviposition pots, hereafter called “ovipots”) with a hole at the bottom filled with a 2-cm layer of plaster to maintain high humidity without water condensation. The container was closed by a lid with a net to prevent larvae from escaping and to allow adult feeding (and potentially ovipositing) on a slice of apple placed on the top. During ovipot servicing, flies were temporarily put in an empty cage of 30 cm^3^. Checks were performed every 2 days, when the piece of apple was changed and eggs/larvae potentially present on it were gently transferred using a dissection needle into a 10-ml container filled with a solid medium for hatching and larval development.

Two different solid media were used: Standard (84.3% water, 6.6% yeast, 4.4% sucrose, 0.7% agar, 3.3% cornmeal flour, and 0.7% propionic acid) [21] and Chestnut (84.3% water, 6.6% yeast, 4.4% sucrose, 0.7% agar, 2.6% chestnut flour, 0.7% banana, and 0.7% propionic acid).

Additionally, adult feeding was enriched by a liquid dietary supplement (i.e. 77.7% distilled water, 19.4% snail extract-based syrup—Siromuicil, Herbit Italia Srl—and 2.9% sodium chloride) soaking a cotton wool inside the pot without using chemicals against moulds.

Ovipot moisture was regularly provided, wetting the plaster as needed, contemporarily avoiding excess of water and the consequent development of moulds. Sibling puparia were transferred and pooled in another plastic container in dry conditions during the entire pupation period in a plastic box and discarded if no adults emerged after 30 days. Adult progeny of a single wild female was kept in pool at the same parental conditions (maximum 10 flies/ovipot) maintaining both sexes in the same container to allow mating. All ovipots were stored in large plastic boxes (50 × 80 × 40 cm) placed in a climatic chamber with a photoperiod of 14:10 h light:dark, 26 ± 2 °C and 80 ± 10% RH.

### Statistical analysis

As only *P. oldenbergi* and *P. variegata* are known to be vectors of *Thelazia callipaeda*, focus was placed on these two species, as no production of progeny was obtained for *P. semivirgo.*

A negative binomial generalized linear model was performed to test the differences in oviposition rates between field-collected females of *P. oldenbergi* and *P. variegata*, as follows:

$$\log y_{i} = \beta_{j} X + \varepsilon_{i}$$

where $y_{i}$ is the oviposition rate for the *i*th pot and $\beta$ is the effect of the *j*th species, with *j* representing a factor with two levels (*P. oldenbergi* and *P. variegata*) [22].

Additionally, the same model structure using a linear model analysis was carried out to test puparia production of *P. oldenbergi* and *P. variegata* according to artificial diets. In this case, $y_{i}$ is the puparia rate (n. puparia/females/pot) for the *i*^th^ pot and $\beta$ is the effect of the *j*^th^ artificial diet, with *j* representing a factor with two levels (chestnut and standard media).

Kaplan-Meier analysis was carried out to determine the survival probability of *Phortica* puparia and adults. The survival probability at time *t*_*i*_, *S* (*t*_*i*_) is calculated as follows:

$$S\,\left( {t_{i} } \right) = S\,\left( {t_{i} - 1} \right) \times \left( {1 - d_{i} /n_{i} } \right)$$

where *S* is survival, *t*_*i*_ is time, *d*_*i*_ is the number of events, and *n*_*i*_ is the number of flies alive just before *t*_*i*_ [23].

To test the robustness of the analysis, a log-rank test was performed approximately distributed as a chi-square function.

## Results

### *Phortica* spp. development and reproduction parameters

Field-collected *Phortica* spp. females (*n* = 130; *P. oldenbergi* = 71; *P. semivirgo* = 3; *P. variegata* = 56) left to singularly oviposit in the plastic containers led to the production of three generations (F1 = 783; F2 = 109; F3 = 6) (Table 1). The oviposition rates for field-collected females (based on mean number of eggs oviposited per female) were significantly higher for *P. oldenbergi* compared to *P. variegata* (negative binomial GLM; *Z* = − 2.637, *P* = 0.008; Table 1). Among F2 specimens, only those belonging to *P. oldenbergi* were able to produce F3 eggs. When comparing the mean number of puparia and adults per female in F1 and F2 generations, the highest value was reached by *P. oldenbergi* followed by *P. variegata* (Table 1). The mean number of development days from puparia to adults (pupation) varied from a minimum of 12 ± 1 (range 4–49 ± 1) in *P. oldenbergi* F1 to a maximum of 20 ± 1 (range 11–20 ± 1) in F3 of the same species. Sex ratio of F1 was slightly unbalanced in favour of females along the sampling season for both *P. variegata* and *P. oldenbergi* with an average value of female proportions of 56% (Table 2).

**Table 1:** Average values of eggs, puparia and adult progeny obtained from field-collected (WF), F1 and F2 females of *Phortica oldenbergi*, *Phortica semivirgo* and *Phortica variegata* according to feeding medium (Chestnut, Standard)

Species	Medium	Parental generation				F1 generation				F2 generation			
		Wild females	Eggs/WF	Puparia/WF	Adults/WF	F1 females	Eggs/F1	Puparia/F1	Adults/F1	F2 females	Eggs/F2	Puparia/F2	Adults/F2
*Phortica oldenbergi*	Chestnut	4	48.2 (min: 1, max: 41, SD: 8.7)	33.8 (min: 1, max: 19, SD: 4.9)	13.5 (min: 1, max: 79, SD: 13.2)	61	1.4 (min: 1, max: 30, SD: 3.2)	0.3 (min: 2, max: 6, SD: 0.7)	0.1 (min: 3, max: 13, SD: 2.4)	13	0	0	0
	Standard	67	33.0 (min: 1, max: 69, SD: 10.8)	8.2 (min: 1, max: 19, SD: 3.2)	2.9 (min: 1, max: 31, SD: 4.1)	189	5.9 (min: 1, max: 42, SD: 5.8)	0.7 (min: 1, max: 9, SD: 1.2)	0.3 (min: 1, max: 25, SD: 2.1)	43	3.2 (min: 1, max: 18, SD: 3.2)	0.2 (min: 1, max: 2, SD: 0.3)	0.1 (min: 1, max: 2, SD: 0.7)
*Phortica semivirgo*	Standard	3	63.7 (min: 1, max: 19, SD: 6.7)	27.3 (min: 1, max: 12, SD: 2.9)	7.0 (min: 4, max: 23, SD: 4.6)	21	10.8 (min: 1, max: 33, SD: 4.9)	0	0	0	0	0	0
*Phortica variegata*	Chestnut	6	11.9 (min: 1, max: 12, SD: 3.2)	9.2 (min: 1, max: 5, SD: 2.1)	4.5 (min: 1, max: 17, SD: 4.3)	19	1.8 (min: 1, max: 15, SD: 2.2)	0	0	0	0	0	0
	Standard	50	23.8 (min: 1, max: 44, SD: 5.6)	6.8 (min: 1, max: 11, SD: 1.8)	1.8 (min: 1, max: 29, SD: 2.6)	85	1.3 (min: 1, max: 10, SD: 1.3)	0.01 (min–max: 1, SD: 0.06)	0.01 (min–max: 1, SD: 0.05)	1	0	0	0

**Table 2:** Proportion of F1 female progeny obtained from field-collected females of *Phortica variegata* and *Phortica oldenbergi* according to the sampling day

Collection date	*Phortica variegata* (%)	*Phortica oldenbergi* (%)
28/05/2020	60	72
04/06/2020	50	na
12/06/2020	53	47
19/06/2020	59	52
02/07/2020	43	61
09/07/2020	49	50
13/08/2020	66	na
27/08/2020	62	46
03/09/2020	44	88
10/09/2020	76	39
15/09/2020	na	48
Mean proportion	56	56

*na* no progeny was obtained

### Influence of artificial diets on *Phortica* spp. breeding

**Fig. 1:**
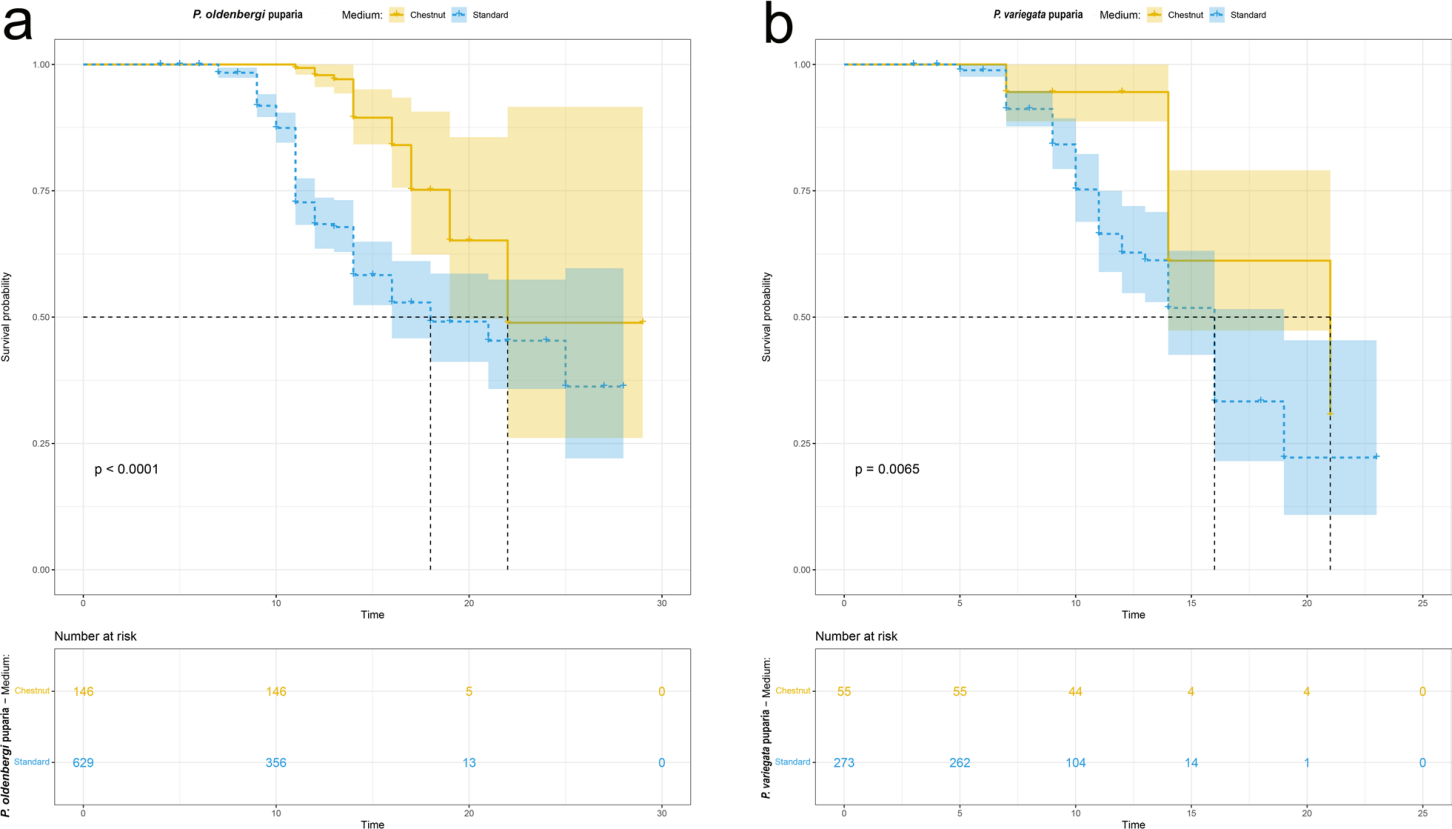
Survival probability curves of *Phortica oldenbergi* (**a**) and *Phortica variegata* (**b**) puparia treated with different media (Chestnut,Standard)

**Fig. 2:**
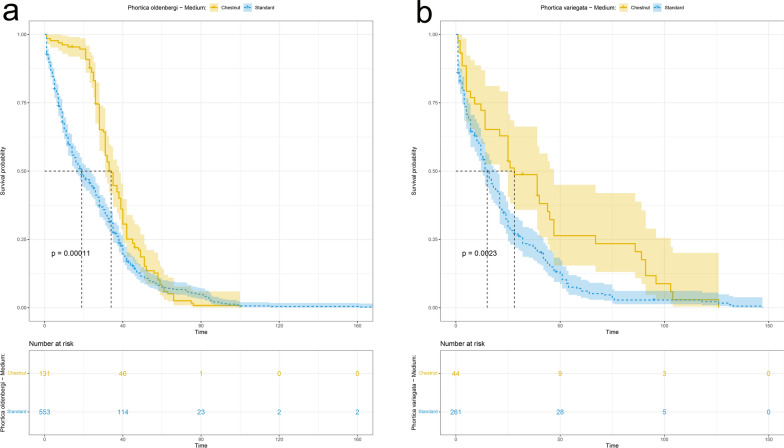
Survival probability curves of *Phortica oldenbergi* (**a**) and *Phortica variegata* (**b**) adults treated with different media (Chestnut, Standard)

The chestnut rearing medium led to a higher production of *P. oldenbergi* F1 adults compared to standard medium (4.6:1; negative binomial GLM: *Z* = − 2.940, *P* = 0.003). Conversely, no significant difference for *P. variegata* F1 adults was obtained between the two rearing media (2.5:1; negative binomial GLM: *Z* = − 1.19, *P* = 0.23; Table 1). Comparing the two feeding media, the average development time of *P. oldenbergi* progeny was lower for larvae fed with the standard medium (F1: 12 ± 1 days; F2: 15 ± 1 days) compared with chestnut medium (F1: 14 ± 1 days; F2: 23 ± 1 days). Conversely, F1 development of *P. variegata* showed no difference between the two media (13 ± 1 days with both feeding conditions; Table 3). The survival probability of chestnut-reared puparia of *P. oldenbergi* was higher compared to the standard-reared ones (log-rank test: χ^2^ = 39.1; *P* < 0.0001; Fig. 1a). These results indicate that chestnut medium favoured pupal development of this species (median survival time for chestnut and standard: 22 and 18 days, respectively). Accordingly, also survival curves for *P. variegata* puparia showed significant difference in the use of the two media (log-rank test: χ^2^ = 7.4, *P* = 0.007; median survival time of 21 days in chestnut-reared puparia and 16 days in standard-reared puparia, Fig. 1b). Likewise, the survival probability curves of *P. oldenbergi* adults showed a significant difference between the two media, with the median survival time of the chestnut-reared flies that is almost doubled compared to the standard-reared ones (34 and 19 days, respectively; log-rank test: χ^2^ = 14.9; *P* = 0.0001; Fig. 2a). Similarly, survival probability is also higher for *P. variegata* chestnut-reared adults (chestnut: 28 days, standard: 15 days; log-rank test: χ^2^ = 9.3; *P* = 0.002; Fig. 2b).

**Table 3:** Mean time of development from egg to adult (days ± 1) of *Phortica oldenbergi*, *Phortica semivirgo* and *Phortica variegata* per generation (F1, F2, F3) and feeding medium (Chestnut, Standard)

Species	Medium	F1 (days ± 1)	F2 (days ± 1)	F3 (days ± 1)
*Phortica oldenbergi*	Chestnut	14 (min = 11, max = 33, SD = 2.7)	23 (min = 16, max = 30, SD = 6.1)	na
	Standard	12 (min = 4, max = 49, SD = 5.9)	15 (min = 4, max = 30, SD = 7.2)	20 (min = 11, max = 30, SD = 8.6)
*Phortica semivirgo*	Standard	15 (min = 7, max = 21, SD = 8.3)	na	na
*Phortica variegata*	Chestnut	13 (min = 7, max = 21, SD = 3.3)	na	na
	Standard	13 (min = 3, max = 46, SD = 9)	12	na

*na* data not available

## Discussion

The breeding protocol presented here indicates the importance of optimizing parameters such as adult density and diet in successfully breeding *Phortica* species. In fact, a F3 has been obtained for the first time, with a substantial improvement compared to previous attempts [19]. To date, a single laboratory breeding protocol for *P. variegata* has been described [19], in which the authors successfully bred this species up to the second generation adopting a simple approach based on feeding ad libitum with fresh apple at all development stages of the flies in a 30-cm^3^ cage with high relative humidity. Compared to previous protocols [19], in which 0.5 F1 adults/females were obtained, this study shows that limiting the fly numbers per pot, the modulation of relative humidity during the life cycle, with drier conditions for pupal phase, and optimal feeding based on chestnut flour and liquid dietary supplement provide higher performance in terms of adult progeny for both F1 and F2 (*P. variegata*: 4.5 F1/f, 1.8 F2/f; *P. oldenbergi*: 13.5 F1/f, 1.4 F2/f; Table 1). The unequal number of females tested with chestnut medium compared with the standard one was a consequence of its late introduction in the rearing protocol. It was impossible to reach equal numbers of flies for both media as the season ended and field flies were no longer available.

Moreover, this rearing protocol allowed breeding *P. oldenbergi* for the first time to our knowledge, providing first data on its life history traits. Also, this species has been demonstrated to be more adapted to insectary conditions compared to *P. variegata*, encouraging its employment as a potential model for challenge studies and trials of veterinary products.

The assumption that *P. variegata* is closely associated with oak forests [24], where acorns or other nuts can be one of the potential feeding sources for larvae (J. Jaenike, personal communication), might not be acceptable for this species over its whole areal, as oak species are rare to absent in central and northern Europe. However, several nut tree species are widely distributed in the southern part of the European continent, particularly in Italy, where *P. variegata* is diffused and *P. oldenbergi* was detected (Bernardini et al*.*, unpublished data). Therefore, a chestnut-based medium could approximate natural feeding conditions, with better performance than a standard *Drosophila* medium based on corn flour, but also compared to fresh fruit. In addition, the liquid dietary supplement containing sodium chloride and mucin proteins (snail extract) might have partially compensated the deficiency of salts and proteins consequent to the lack of lachryphagy under laboratory breeding conditions. Lachryphagous behaviour is described in several Steganinae species other than those belonging to *Phortica* genus, in particular *Amiota*, *Gitona*, *Paraleucophenga* and *Apenthecia* [1]. However, this is not a peculiar behaviour of drosophilid flies, as other Diptera (e.g. Muscidae, Fanniidae, Chloropidae and Paraleucopidae) show some degree of lachryphagy in both sexes, with a prevalence of females as opposed to males of Steganinae [1]. Also several Lepidoptera show a lachryphagous behaviour in males and occasionally also in females [1]. Finally, this behaviour was observed in cockroaches exploiting lacrimal secretions from reptiles [25]. This supplementary feeding seems to be useful to obtain essential compounds, such as minerals, urea, glucose and proteins, possibly useful to increase fitness of lachryphagous species [25].

Despite these encouraging results, the low initial number of wild females used in this study, especially the low number reared with the chestnut medium, might have affected the possibility to obtain a stable colony. This may be a consequence of the low initial genetic variability of the laboratory population [26] as well as an intrinsic low oviposition rate of the field-collected *Phortica* spp. (*P. oldenbergi*: 33.9 eggs/female, *P. variegata*: 22.6 eggs/female). Comparing data herein obtained about oviposition rates with those of species belonging to the *Drosophila* genus (> 2500 eggs/female; [27]), the low progeny numbers per generation may account for the biological limitation of *Phortica* spp. in obtaining a stable colony. New attempts will be conducted with a higher starting number of field-collected females to overcome these limitations and try to obtain a stable colony.

Data also allowed clarifying an open question about the population dynamics of *P. variegata* in the field. In fact, it is known that this species shows a switch of sex ratio along the breeding season, with an increase in lachryphagous males during late summer [28]. The F1 obtained by field-collected females from May to October did not indicate any shift in progeny sex ratio along the season (Table 2). This led to the conclusion that the switch of relative proportion of males during the season is a consequence of a sampling bias due to their feeding behaviour instead of a physiological change of sex ratio in the population.

## Conclusions

This novel breeding protocol of *Phortica* spp. allowed to investigate aspects of life history traits of these drosophilids, which are involved in the transmission of the zoonotic eyeworm *T. callipaeda*. Controlled climatic parameters and fly densities, together with the introduction of a more proper feeding medium (i.e. considering the needs of *Phortica* spp. associated to oak forests) significantly improved the survival and fecundity of both *P. variegata* and *P. oldenbergi*. This standardized approach allowed to reach F3 generation for the first time, representing the basis for the establishment of stable colonies, which are an essential requirement for future behavioural/physiological studies on these vectors as well as pharmaceutical trials of veterinary and medical products.

## Data Availability

The data that support the findings of this study are available from the corresponding author, MP, upon reasonable request.

## Abbreviations

RH: Relative humidity
GLM: Generalized linear model
F1: First generation
F2: Second generation
F3: Third generation
